# Primary ciliary dyskinesia as a rare cause of male infertility: case report and literature overview

**DOI:** 10.1186/s12610-024-00244-z

**Published:** 2024-12-18

**Authors:** Jan Novák, Lenka Horáková, Alena Puchmajerová, Viktor Vik, Zuzana Krátká, Vojtěch Thon

**Affiliations:** 1 Department of Urology, General University Hospital and First Faculty of Medicine, Charles University, Prague, Czech Republic; 2 Urology and Andrology, IVF Clinic GENNET, Prague, Czech Republic; 3 Clinical Genetics, IVF Clinic GENNET, Prague, Czech Republic; 4 Transplantation Surgery Department, Institute for Clinical and Experimental Medicine, Prague, Czech Republic; 5 Laboratory of Immunology, IVF Clinic GENNET, Prague, Czech Republic; 6 RECETOX, Faculty of Science, Masaryk University, Brno, Czech Republic

**Keywords:** Primary ciliary dyskinesia, Kartagener’s syndrome, Immotile sperm, Infertility, Dyskinésie ciliaire primitive (DCP), Syndrome de Kartagener, Spermatozoïdes immobiles, Infertilité

## Abstract

**Background:**

Primary ciliary dyskinesia (PCD) is a heterogenous disease caused by mutations of miscellaneous genes which physiologically play an important role in proper structure and/or function of various cellular cilia including sperm flagella. Besides male infertility, the typical phenotypes, based on decreased mucociliary clearance, are lifelong respiratory issues, i.e., chronic bronchitis leading to bronchiectasis, chronic rhinosinusitis, and chronic otitis media. Moreover, since motile cilia are important during embryological development in the sense of direction of gut rotation, 50% of affected individuals develop situs inversus – so-called Kartagener’s syndrome.

**Case presentation:**

We present two cases of PCD as a rare cause of male infertility.

**Conclusions:**

Primary ciliary dyskinesia should be suspected in infertile males having (sub)normal sperm concentration values with persistent zero motility together with patient’s and/or family history of respiratory symptoms like bronchiectasis, chronic cough, rhinitis, recurrent sinusitis, and otitis media. Due to more than 50 identified mutations until now, the causal mechanism of male infertility is miscellaneous and not in all cases known in detail. Besides impaired sperm motility, other mechanisms significantly decreasing efficacy of assisted reproduction techniques play a pivotal role. Thus, proper diagnostic work-up including, among others, sperm DNA fragmentation, is mandatory to avoid ineffective treatment burden.

## Resume

La dyskinésie ciliaire primitive (DPC) est une maladie génétique rare causée par des mutations impactant des gènes essentiels à la structure et/ou au fonctionnement du flagelle spermatique et des cils mobiles qui sont présents à la surface de plusieurs types cellulaires. Outre l'infertilité masculine, les phénotypes typiques de cette pathologie sont la diminution de la clairance mucociliaire associée à des contextes récurrents d’otite, de rhinosinusite et de bronchite chronique pouvant conduire à une bronchectasie. De plus, comme les cils mobiles sont importants au cours du développement embryonnaire pour mettre en place la latéralisation des organes, 50 % des individus affectés développent un *situs inversus*, aussi appelé syndrome de Kartagener.

Nous présentons deux cas de PCD comme cause rare d'infertilité masculine pour lesquels nous avons identifié des mutations génétiques causales. Nous discutons le diagnostic de la PCD qui doit être suspectée chez les hommes infertiles ayant des valeurs de concentration de spermatozoïdes sub-normales avec une motilité nulle persistante ainsi que des antécédents de symptômes respiratoires. Malgré l’identification de plus de 50 mutations causales, à ce jour, les mécanismes physiopathologiques de l'infertilité masculine restent très peu définis. Dans les situations d’infertilité en contexte PCD, en plus de l’altération de la mobilité des spermatozoïdes, des éléments de la littérature suggèrent que d’autres défauts pourraient potentiellement altérer l’efficacité des techniques de procréation. Ainsi, nous recommandons un bilan diagnostique incluant, entre autres, le test de fragmentation de l’ADN des spermatozoïdes, afin de maximiser les chances d’efficacité des techniques d’assistance à la procréation.

## Introduction

Primary ciliary dyskinesia (PCD) is a heterogenous disease caused by mutations of miscellaneous genes which physiologically play an important role in proper structure and/or function of various cellular cilia including sperm flagella.

Besides male infertility, the typical phenotypes, based on decreased mucociliary clearance, are lifelong respiratory issues, i.e., chronic bronchitis leading to bronchiectasis, chronic rhinosinusitis, and chronic otitis media. Moreover, since motile cilia are important during embryological development in the sense of direction of gut rotation, 50% of affected individuals develop situs inversus – so-called Kartagener’s syndrome [1]. Less frequent symptoms are complex congenital heart disease, hydrocephalus, and retinitis pigmentosa.

As mentioned above, since PCD is a heterogenous disease with variable clinical appearance, some of the individuals, especially those with less pronounced respiratory issues, may miss their correct diagnosis usually given already in childhood. There are no data describing the prevalence of PCD among infertile couples. However, given the fact that the reported incidence is approximately 1 in 10.000–20.000 live births [2, 3] and most of the patients are diagnosed by paediatricians – median age at diagnosis is 5,3 years, and, in cases of situs inversus, as early as 3,5 years [4] – one may expect the prevalence among infertile couples to be significantly lower.

We present two cases of PCD as a rare cause of male infertility, and a literature overview.

## Case presentation

### Case 1

A 38-year-old male patient from an infertile couple has been referred to an andrologist. He has tried to conceive for last 2 years with his 40-year-old female partner. She has never been pregnant, she has not undergone any infertility treatment, and apart from her age, she has had normal gynaecological finding.

Two baseline sperm analyses have been performed with similar results: sperm concentration at the lower reference level, but 100% of the sperm have been immotile and thus evaluated as necrozoospermia (Table 1).

**Table 1:** Case 1 – sperm analyses performed initially (Sperm analysis 1 and 2, respectively) and after treatment with clomiphene citrate 50 mg once a day (Sperm analysis 3)

	Ref. values [5]	Sperm analysis 1	Sperm analysis 2	Sperm analysis 3
Abstinence delay [days]	2–7	5	5	14
Semen volume [ml]	> 1,4	3	4,5	8
Sperm concentration [10^6^/ml]	> 16	15	14	33
Total sperm count [10^6^]	> 39	45	63	264
Total sperm motility [%]	> 42	0	0	0
Normal sperm morphology [%]	> 4	0	0	0

Since his childhood, he has been followed up by a pneumologist and an immunologist for bronchiectasis, bronchial asthma, chronic cough, and rhinitis. Chronically, he has been taking many drugs – salmeterol/fluticasone spray, salbutamol, sertraline, erdosteine, fluticasone furoate spray, transfer factors from porcine spleen, pancreatic enzymes, and lansoprazole.

Neither physical examination nor ultrasonography have revealed any abnormality. Blood analysis has showed normal levels of luteinizing hormone (1,59 U/l), prolactin (166,95 mIU/l), thyroid-stimulating hormone (2,21 mU/l), sex hormone-binding globulin (34,7 nmol/l), but elevation of follicle-stimulating hormone (10,8 U/l) and decrease of testosterone (9,22 nmol/l) have been apparent.

Clomiphene citrate 50 mg once a day has led to increase in luteinizing hormone (11,42 U/l), follicle-stimulating hormone (25,01 U/l), and testosterone (53,03 nmol/l) levels.

Sperm analysis has shown an increase in sperm concentration, however, still 100% of sperm have been immotile (Table 1). Additional cytometric sperm analysis (ApoFlowEx^®^ FITC kit by Exbio Praha, a. s.) using Annexin V – FITC (fluorescein isothiocyanate) and PI (propidium iodide) as markers has revealed 27,2% vital, 8% early apoptotic, and 64,8% late apoptotic and necrotic sperm. Sperm DNA fragmentation measured by TUNEL (terminal deoxynucleotidyl transferase dUTP nick end labelling; Apo Direct™ by Phoenix Flow Systems) has been 52,4% – normal value of sperm DNA fragmentation measured by this method is below 20%.

His karyotype has been 46,XY, and neither CFTR (cystic fibrosis transmembrane conductance regulator) gene mutation nor chromosome Y microdeletions have been detected. Among 836 mutations tested by NGS (next-generation sequencing) CarrierTest™, a mutation p.Gln799Glu/p.Gln799glu in the androgen receptor gene causing mild androgen insensitivity has been found.

Based on his lifelong respiratory issues and persistent zero sperm motility, PCD has been suspected. Additional NGS DNA analysis of 484 genes, of which 38 (CCDC39, CCDC40, CCDC65, CCDC103, CCDC114, CCNO, CENPF, CFAP300, DAW1, DNAAF1, DNAAF2, DNAAF3, DNAAF4, DNAAF5, DNAH1, DNAH3, DNAH5, DNAH8, DNAH9 (= DNAL1), DNAH11, DNAI1, DNAI2, DNAJB13, DRC1, GDF1, LRRC6, MCIDAS, NEK4, NME8, PIH1D3, RSPH1, RSPH3, RSPH4A, RSPH9, SPAG1, STK36, TTC25, ZMYND10) potentially cause PCD, has shown a pathogenic homozygous sequence c.2014C > T in SPAG1 gene with autosomal recessive inheritance and thus causing PCD that results in male infertility [6].

In case of severe asthenozoospermia caused by PCD, IVF (in-vitro fertilization) in combination with ICSI (intracytoplasmic sperm injection), including PGT-A (preimplantation genetic testing of aneuploidy) might be a treatment option. However, based on the female partner’s age and high sperm DNA damage – both being strong negative prognostic outcome factors [7], the couple has decided not to proceed with any of the assisted reproductive technologies and has thus remained childless.

### Case 2

A 35-year-old male from an infertile couple has been referred to an andrologist. He has tried to conceive for last 3,5 years with his 31-year-old female partner. She has never been pregnant, but recently she underwent hormonal stimulation with a result of seven fertilized oocytes, however with no pregnancy after frozen embryo transfers. Besides these, she has had normal gynaecological finding.

The sperm analysis has shown excellent sperm concentration, but 100% immotile sperm (Table 2). Analogous results were obtained previously at other andrological facilities, but until now with no explanation of the cause.

**Table 2:** Case 2 – baseline sperm analysis

	Ref. values [5]	Sperm analysis
Abstinence delay [days]	2–7	3
Semen volume [ml]	> 1,4	2,5
Sperm concentration [10^6^/ml]	> 16	70
Total sperm count [10^6^]	> 39	175
Total sperm motility [%]	> 42	0
Normal sperm morphology [%]	> 4	4

Since his childhood, he has suffered from many episodes of otitis media (approx. 40). Moreover, he has got chronic rhinitis, and he has been treated for psoriasis. He is not using any chronic medication.

Neither physical examination nor ultrasonography have revealed any abnormality. Blood analysis has shown normal levels of luteinizing hormone (0,91 U/l), follicle-stimulating hormone (2,1 U/l), prolactin (197,92 mIU/l), thyroid-stimulating hormone (2,92 mU/l), sex hormone-binding globulin (23,1 nmol/l), but mild low testosterone (10,88 nmol/l) level has been apparent.

Clomiphene citrate 50 mg once a day has led to an increase in luteinizing (2,72 U/l), follicle-stimulating hormone (5,16 U/l), and testosterone (32,26 nmol/l) levels.

Additional cytometric sperm analysis (ApoFlowEx^®^ FITC kit by Exbio Praha, a. s.) using Annexin V – FITC and PI as markers has revealed 72% vital, 6,7% early apoptotic, and 21,3% late apoptotic and necrotic sperm. Sperm DNA fragmentation measured by TUNEL (Apo Direct™ by Phoenix Flow Systems) has been as low as 3,2%.

His karyotype is 46,XY, and neither CFTR gene mutation nor chromosome Y microdeletions have been detected.

From the patient’s family history, his brother is suffering from chronic cough. Additionally, the patient’s maternal uncle and male cousin were diagnosed with situs inversus.

Based on the patient’s lifelong susceptibility to recurrent otitis media, persistent zero sperm motility, and presence of two individuals in his family having situs inversus, PCD has been suspected.

The MLPA (multiplex ligation-dependent probe amplification) method hasn’t found any intragenic mutation in selected regions of DNAI1 and DNAH5 genes. However, CNV (copy number variation) analysis of 574 genes, of which 41 (CCDC39, CCDC40, CCDC65, CCDC103, CCDC114, CCNO, CENPF, CFAP300, DAW1, DNAAF1, DNAAF2, DNAAF3, DNAAF4, DNAAF5, DNAH1, DNAH3, DNAH5, DNAH8, DNAH9 (= DNAL1), DNAH11, DNAI1, DNAI2, DNAJB13, DRC1, GAS2L2, GAS8, GDF1, LRRC6, LRRC56, MCIDAS, NEK4, NME8, PIH1D3, RSPH1, RSPH3, RSPH4A, RSPH9, SPAG1, STK36, TTC25, ZMYND10) cause PCD, has shown a hemizygous exon 3 deletion of DNAAF6 (dynein axonemal assembly factor 6, previously named PIH1D3) gene, with X-linked recessive inheritance [8, 9]. Since MLPA probes for the DNAAF6 gene are not available, the precise verification of the above-mentioned mutation is currently not feasible. The deletion has been described as a possible cause of PCD that affects males. Females are usually asymptomatic carriers of the mutation; however, they may develop a wide range of symptoms based on their variable X-chromosome inactivation (XCI) pattern [10].

The couple has been advised to undergo second hormonal stimulation. A total of 13 oocytes have been obtained from which seven have been fertilized and only one has developed properly. After frozen embryo transfer the female partner has become pregnant and has given birth to a full-term healthy boy.

## Discussion

PCD is a rare condition with estimated incidence of 1 in 10.000–20.000 live births [2, 3]. However, the real prevalence is expected to be higher given misdiagnosis due to lack of awareness. The worldwide prevalence, based on the frequency of most common causative genes, was recently calculated to be approx. 1 in 7.500 [11]. The prevalence in some consanguineous communities may reach up to 1 in 400 live births [12].

The phenotype, typically chronic bronchitis leading to bronchiectasis, chronic rhinosinusitis, and chronic otitis media together with male infertility, is given by ciliary dysfunction. If situs inversus, which is found in 50% of affected individuals, is present, the nosological entity is called Kartagener’s syndrome.

Cilia and sperm flagella share the same basic inner structure, i.e. an axoneme consisting of two central microtubules surrounded by nine peripheral microtubular doublets, so-called „9 + 2 structure“ [13]. The peripheral doublets are circumferentially interconnected by nexin links, and there are radial spokes connecting them with the central pair of microtubules. The active movement of these tubular structures is enabled by molecular motors, i.e. inner and outer dynein arms [14], whose coordination is ensured by nexin-dynein regulatory complexes.

Despite the above-mentioned shared inner structure, there are some distinct differences between cilia and sperm flagella [15]. In comparison to cilia, sperm flagella are up to 5- to 10-fold longer and might be divided into three parts: midpiece, principal piece, and end piece, each with a distinct structure [13]. Additionally, sperm flagella contain outer dense fibres, fibrous sheath, and mitochondria.

Until now, more than 50 genes [13] whose mutations cause PCD, with most commonly autosomal recessive inheritance pattern (two most studied genes being DNAI1 a DNAH5) [14, 16], have been identified. Many of them affect formation and/or function of dynein arms, but the exact molecular mechanism in most mutations is not known and further research is necessary. Some studies, however, suggest there are differences in dyneins and other proteins presented in cilia and sperm flagella, possibly explaining the variable presence of infertility and respiratory symptoms in individuals affected by PCD [13, 17] – e.g. CCDC114 gene mutations usually do not lead to infertility due to low gene expression in testicles [12].

SPAG1, mentioned in the first case report, is a gene that plays an essential role in the cytoplasmic assembly and/or trafficking of the axonemal dynein arms [18]. The diagnosed mutation c.2014C > T (p.Gln672*; allele frequency 7,62 × 10^–5^) in SPAG1 gene is a nonsense mutation creating a premature termination codon that results in an absent or disrupted protein product [19] and is widely spread (accounting for approx. 14% PCD patients) in the Slavic region including Poland, Czech Republic, and Slovakia [20].

In the second case report, DNAAF6, formerly known as PIH1D3, is a protein-coding gene with X-linked recessive inheritance and is responsible for early axonemal dynein arm assembly. Mutations (deletions of exons 1–5, 2–5, 6, and 8, respectively) of the gene have been recently identified as a possible cause of PCD, namely causing the absence of outer and inner dynein arms in cilia and sperm flagella [21–23]. To our knowledge, isolated deletion of exon 3 of DNAAF6 gene as a cause of PCD has not previously been published and its exact pathogenic mechanism is unknown.

Nevertheless, ICSI remains literally the only option for affected males to get biological offspring. Selection of a single viable sperm among immotile sperm is sometimes challenging, and various methods like the hypoosmotic swelling test [24] or laser-assisted immotile sperm selection (LAISS) might be used.

According to some studies, male fertile potential is not only affected by decreased sperm motility, but other mechanisms like ciliary dysfunction of the reproductive tract (most importantly efferent ductules that connect rete testis with the epididymis), which affects both spermatogenesis and subsequent sperm transport, seem to be involved in some mutations [13]. However, some recent studies suggest that the cilia in efferent ductules can prevent sperm aggregation rather than playing role in sperm transport per se [11]. Conventional sperm analysis usually shows normal or subnormal sperm count, but very low or even zero total motility in consecutive occasions. Azoospermia is not an exception, especially in mutations causing ciliary dysfunction in efferent ductules.

Additionally, mutations affecting centriole function seem to hamper pronucleus formation due to mitotic spindle failure resulting in inhibited cell division. This might explain why, despite expectations [24], IVF combined with ICSI is ineffective in some individuals [25]. Similar consequences may occur for sperm ultrastructural abnormalities, like fibrous sheath deformity that results in zero mitochondrial activity, which could be revealed by electron microscopy [1].

Genetic abnormalities (e.g., DNA repair gene mutations, mitochondrial DNA mutations increasing oxidative stress, mutations affecting spermatogenesis or cell division in general) are listed as possible causes of increased sperm DNA damage that can lead to male infertility [26–29]. However, there are only three case reports available concerning sperm DNA fragmentation in PCD patients, and these have conflicting results [1, 30, 31]. In couples, who failed in ICSI treatment, increased sperm DNA fragmentation was observed. Since the presence of somatic cells in ejaculate that produce reactive oxygen species that cause lipid peroxidation is rather unlikely, other mechanisms including ultrastructural sperm abnormalities that affect proper nuclear condensation might be involved. However, this must be confirmed by further research [1]. For that reason, sperm DNA fragmentation evaluation should be made in PCD patients before infertility treatment [1, 13]. Possible reversible causes of increased sperm DNA fragmentation should be eliminated prior ICSI. If no treatable factors are apparent, empiric antioxidant treatment is an option. In cases of persistent increased sperm DNA fragmentation despite the above-mentioned measures, testicular sperm extraction might yield better quality sperm, as recommended by the Guidelines of the European Association of Urology [7].

In females, PCD may increase the risk of ectopic pregnancy due to immotile cilia of the Fallopian tube lining [16, 32]. However, smooth muscular contractions seem to play a more important role in oocyte transportation than coordinated ciliary movement [16].

## Conclusion

Primary ciliary dyskinesia should be suspected in infertile males having (sub)normal sperm concentration values with persistent zero motility. This should be assessed together with the patient’s and/or family history of respiratory symptoms like bronchiectasis, chronic cough, rhinitis, recurrent sinusitis, and otitis media. Additionally, situs inversus is found in 50% of affected individuals. Since more than 50 mutations have been described, the causal mechanism of male infertility is miscellaneous and not in all cases known in detail. Besides impaired sperm motility, other mechanisms that decrease the efficacy of assisted reproduction techniques likely play a pivotal role. Thus, a proper diagnostic work-up, including among others, assessment of sperm DNA fragmentation, is mandatory to avoid ineffective treatment burden.

## Data Availability

The datasets used and/or analysed during the current study are available from the corresponding author on reasonable request.

## Abbreviations

CFTR: Cystic fibrosis transmembrane conductance regulator
CNV: Copy number variation
DNA: Deoxyribonucleic acid
DNAAF6: Dynein axonemal assembly factor 6
FITC: Fluorescein isothiocyanate
ICSI: Intracytoplasmic sperm injection
IVF: In-vitro fertilization
LAISS: Laser-assisted immotile sperm selection
MLPA: Multiplex ligation-dependent probe amplification
NGS: Next-generation sequencing
PCD: Primary ciliary dyskinesia
PI: Propidium iodide
PGT-A: Preimplantation genetic testing of aneuploidy
TUNEL: Terminal deoxynucleotidyl transferase dUTP nick end labelling
XCI: X-chromosome inactivation

## References

[1] Pariz JR, Rané C, Drevet J, Hallak J. Dysplasia of the fibrous sheath with axonemal and centriolar defects combined with lack of mitochondrial activity as associated factors of ICSI failure in primary ciliary dyskinesia syndrome. Int Braz J Urol. 2021;47(3):617–26. 33621011 10.1590/S1677-5538.IBJU.2019.0362 PMC7993943

[2] Knowles MR, Zariwala M, Leigh M. Primary Ciliary Dyskinesia. Clin Chest Med. 2016;37(3):449–61. 27514592 10.1016/j.ccm.2016.04.008 PMC4988337

[3] Mirra V, Werner C, Santamaria F. Primary ciliary dyskinesia: an update on clinical aspects, genetics, diagnosis, and future treatment strategies. Front Pediatr. 2017;5. Available from: http://journal.frontiersin.org/article/10.3389/fped.2017.00135/full. 10.3389/fped.2017.00135 PMC5465251 28649564

[4] Kuehni CE, Frischer T, Strippoli MPF, Maurer E, Bush A, Nielsen KG, et al. Factors influencing age at diagnosis of primary ciliary dyskinesia in European children. Eur Respir J. 2010;36(6):1248–58. 20530032 10.1183/09031936.00001010

[5] World Health Organization. WHO laboratory manual for the examination and processing of human semen 6th edition 2021. World Health Organization; 2021.

[6] Boaretto F, Snijders D, Salvoro C, Spalletta A, Mostacciuolo ML, Collura M, et al. Diagnosis of Primary Ciliary Dyskinesia by a Targeted Next-Generation Sequencing Panel. J Mol Diagn. 2016;18(6):912–22. 27637300 10.1016/j.jmoldx.2016.07.002

[7] Salonia A, Bettocchi C, Carvalho J, Capogrosso P, Corona G, Hatzchristodoulou G, et al. EAU guidelines on sexual and reproductive health. Edn. presented at the EAU Annual Congress Paris 2024. Arnhem: EAU Guidelines Office; 2024.

[8] UK10K Rare Group, Olcese C, Patel MP, Shoemark A, Kiviluoto S, Legendre M, et al. X-linked primary ciliary dyskinesia due to mutations in the cytoplasmic axonemal dynein assembly factor PIH1D3. Nat Commun. 2017;8(1):14279. 28176794 10.1038/ncomms14279 PMC5309803

[9] Wang Y, Tu C, Nie H, Meng L, Li D, Wang W, et al. Novel DNAAF6 variants identified by whole-exome sequencing cause male infertility and primary ciliary dyskinesia. J Assist Reprod Genet. 2020;37(4):811–20. 32170493 10.1007/s10815-020-01735-4 PMC7183028

[10] Thomas L, Cuisset L, Papon JF, Tamalet A, Pin I, Abou Taam R, et al. Skewed X-chromosome inactivation drives the proportion of *DNAAF6* -defective airway motile cilia and variable expressivity in primary ciliary dyskinesia. J Med Genet. 2024;61(6):595–604. 38408845 10.1136/jmg-2023-109700

[11] Despotes KA, Zariwala MA, Davis SD, Ferkol TW. Primary Ciliary Dyskinesia: A Clinical Review. Cells. 2024;13(11):974. 38891105 10.3390/cells13110974 PMC11171568

[12] Damseh N, Quercia N, Rumman N, Dell S, Kim R. Primary ciliary dyskinesia: mechanisms and management. Appl Clin Genet. 2017;10:67–74. 29033599 10.2147/TACG.S127129 PMC5614735

[13] Jayasena CN, Sironen A. Diagnostics and Management of Male Infertility in Primary Ciliary Dyskinesia. Diagnostics. 2021;11(9):1550. 34573892 10.3390/diagnostics11091550 PMC8467018

[14] Li P, Sha YW, Ding L. Management of primary ciliary dyskinesia/Kartagener′s syndrome in infertile male patients and current progress in defining the underlying genetic mechanism. Asian J Androl. 2014;16(1):101. 24369140 10.4103/1008-682X.122192 PMC3901865

[15] Sironen A, Shoemark A, Patel M, Loebinger MR, Mitchison HM. Sperm defects in primary ciliary dyskinesia and related causes of male infertility. Cell Mol Life Sci. 2020;77(11):2029–48. 31781811 10.1007/s00018-019-03389-7 PMC7256033

[16] Boon M, Jorissen M, Proesmans M, De Boeck K. Primary ciliary dyskinesia, an orphan disease. Eur J Pediatr. 2013;172(2):151–62. 22777640 10.1007/s00431-012-1785-6

[17] Wallmeier J, Nielsen KG, Kuehni CE, Lucas JS, Leigh MW, Zariwala MA, et al. Motile ciliopathies. Nat Rev Dis Primer. 2020;6(1):77. 10.1038/s41572-020-0209-6 32943623

[18] Knowles MR, Ostrowski LE, Loges NT, Hurd T, Leigh MW, Huang L, et al. Mutations in SPAG1 Cause Primary Ciliary Dyskinesia Associated with Defective Outer and Inner Dynein Arms. Am J Hum Genet. 2013;93(4):711–20. 24055112 10.1016/j.ajhg.2013.07.025 PMC3791252

[19] 8–100233436-C-T | gnomAD v4.1.0 | gnomAD. Available from: https://gnomad.broadinstitute.org/variant/8-100233436-C-T?dataset=gnomad_r4.

[20] Raidt J, Riepenhausen S, Pennekamp P, Olbrich H, Amirav I, Athanazio RA, et al. Analyses of 1236 genotyped primary ciliary dyskinesia individuals identify regional clusters of distinct DNA variants and significant genotype–phenotype correlations. Eur Respir J. 2024;64(2):2301769. 38871375 10.1183/13993003.01769-2023 PMC11306806

[21] Huang C, Liu NC, Wang XB, Gu BH, Zhang JX, Li Z, et al. Novel deletion mutations of the PIH1D3 gene in an infertile young man with primary ciliary dyskinesia and his cousin with Kartagener’s syndrome. Asian J Androl. 2021;23(3):330–2. 33106461 10.4103/aja.aja_43_20 PMC8152414

[22] Xu Y, Ogawa S, Adachi Y, Sone N, Gotoh S, Ikejiri M, et al. A pediatric case of primary ciliary dyskinesia caused by novel copy number variation in PIH1D3. Auris Nasus Larynx. 2022;49(5):893–7. 33812756 10.1016/j.anl.2021.03.012

[23] Paff T, Loges NT, Aprea I, Wu K, Bakey Z, Haarman EG, et al. Mutations in PIH1D3 Cause X-Linked Primary Ciliary Dyskinesia with Outer and Inner Dynein Arm Defects. Am J Hum Genet. 2017;100(1):160–8. 28041644 10.1016/j.ajhg.2016.11.019 PMC5223094

[24] Dávila Garza SA, Patrizio P. Reproductive outcomes in patients with male infertility because of Klinefelter’s syndrome, Kartagener’s syndrome, round-head sperm, dysplasia fibrous sheath, and ‘stump’ tail sperm: an updated literature review. Curr Opin Obstet Gynecol. 2013;25(3):229–46. 23587797 10.1097/GCO.0b013e32835faae5

[25] Newman L, Chopra J, Dossett C, Shepherd E, Bercusson A, Carroll M, et al. The impact of primary ciliary dyskinesia on female and male fertility: a narrative review. Hum Reprod Update. 2023;29(3):347–67. 36721921 10.1093/humupd/dmad003 PMC10152180

[26] Mukherjee S, Ridgeway AD, Lamb DJ. DNA mismatch repair and infertility. Curr Opin Urol. 2010;20(6):525–32. 20852424 10.1097/MOU.0b013e32833f1c21 PMC3079360

[27] Ji G, Long Y, Zhou Y, Huang C, Gu A, Wang X. Common variants in mismatch repair genes associated with increased risk of sperm DNA damage and male infertility. BMC Med. 2012;10(1):49. 22594646 10.1186/1741-7015-10-49 PMC3378460

[28] Shamsi M, Kumar R, Bhatt A, Bamezai RNK, Kumar R, Gupta N, et al. Mitochondrial DNA mutations in etiopathogenesis of male infertility. Indian J Urol. 2008;24(2):150. 19468388 10.4103/0970-1591.40606 PMC2684292

[29] Stavros S, Potiris A, Molopodi E, Mavrogianni D, Zikopoulos A, Louis K, et al. Sperm DNA Fragmentation: Unraveling Its Imperative Impact on Male Infertility Based on Recent Evidence. Int J Mol Sci. 2024;25(18):10167. 39337652 10.3390/ijms251810167 PMC11432134

[30] Cannarella R, Maniscalchi ET, Condorelli RA, Scalia M, Guerri G, La Vignera S, et al. Ultrastructural Sperm Flagellum Defects in a Patient With CCDC39 Compound Heterozygous Mutations and Primary Ciliary Dyskinesia/Situs Viscerum Inversus. Front Genet. 2020;11:974. 33005176 10.3389/fgene.2020.00974 PMC7483550

[31] Nuñez R, López-Fernández C, Arroyo F, Caballero P, Gosálvez J. Characterization of sperm DNA damage in Kartagener’s syndrome with recurrent fertilization failure: Case revisited. Sex Reprod Healthc. 2010;1(2):73–5. 21122600 10.1016/j.srhc.2009.12.001

[32] Zariwala MA, Knowles MR, Leigh MW. Primary Ciliary Dyskinesia. In: Adam MP, Everman DB, Mirzaa GM, Pagon RA, Wallace SE, Bean LJ, et al., editors. GeneReviews®. Seattle (WA): University of Washington, Seattle; 1993. Available from: http://www.ncbi.nlm.nih.gov/books/NBK1122/20301301

